# Adaptive Dynamic Analysis of MEMS Gyroscope Random Noise Based on PID-DAVAR

**DOI:** 10.3390/mi14040792

**Published:** 2023-03-31

**Authors:** Jianing Zhang, Pinghua Li, Zhiyu Yu, Jinghao Liu, Xiaoyang Zhang, Xuye Zhuang

**Affiliations:** School of Mechanical Engineering, Shandong University of Technology, Zibo 255000, China

**Keywords:** MEMS gyroscope, dynamic Allan variance, gyroscope array, Allan variance

## Abstract

As a MEMS gyroscope is susceptible to environmental interference, its performance is degraded due to random noise. Accurate and rapid analysis of random noise of MEMS gyroscope is of great significance to improve the gyroscope’s performance. A PID-DAVAR adaptive algorithm is designed by combining the PID principle with DAVAR. It can adaptively adjust the length of the truncation window according to the dynamic characteristics of the gyroscope’s output signal. When the output signal fluctuates drastically, the length of the truncation window becomes smaller, and the mutation characteristics of the intercepted signal are analyzed detailed and thoroughly. When the output signal fluctuates steadily, the length of the truncation window becomes larger, and the intercepted signals are analyzed swiftly and roughly. The variable length of the truncation window ensures the confidence of the variance and shortens the data processing time without losing the signal characteristics. Experimental and simulation results show that the PID-DAVAR adaptive algorithm can shorten the data processing time by 50%. The tracking error of the noise coefficients of angular random walk, bias instability, and rate random walk is about 10% on average, and the minimum error is about 4%. It can accurately and promptly present the dynamic characteristics of the MEMS gyroscope’s random noise. The PID-DAVAR adaptive algorithm not only satisfies the requirement of variance confidence but also has a good signal-tracking ability.

## 1. Introduction

MEMS gyroscope plays an important role in industrial equipment, inertial navigation systems, and other fields because of their small size and low power consumption [1]. However, the complexity of MEMS gyroscope processing and the variability of the operating environment result in a lot of random noise in the gyroscope’s output signal, which seriously affects the performance of MEMS gyroscopes [2,3]. Therefore, to improve the accuracy of the MEMS gyroscope, it is necessary to comprehensively analyze the random noise characteristics of the MEMS gyroscope and assess its performance. At present, the random error modeling methods of MEMS gyroscopes mainly include the autocorrelation analysis method, power spectral density method, and Allan variance analysis method. Among them, the autocorrelation analysis method requires too much data acquisition time and has a poor analysis effect on error. The power spectral density method is applicable to frequency domain analysis, the power spectral density curves of errors are easy to overlap, making it difficult to separate errors [4,5]. It is worth noting that when the method converts the frequency domain results into time domain characteristics, further analysis and processing lead to a large workload and time-consuming. The Allan variance analysis method is simple in its calculation and can identify noise and quantify the statistical contribution of each kind of noise in random error. The DAVAR method is a combination of Allan variance and window function, it can not only identify the random error of MEMS gyro array, but also show the stability of random error over time. Reference [6] systematically introduced the basic theoretical knowledge of Allan variance modeling inertial sensor error term and expounded the implementation effect in different levels of inertial sensor modeling. Reference [7] proposed a fast DAVAR algorithm in the case of missing data. The results showed that the fast DAVAR method could significantly reduce the calculation time. Reference [8] extended fast DAVAR to discontinuous time series. The improved DAVAR could not only handle discrete data but also consume less computing time. Reference [9]. Proposed an improved fast DAVAR algorithm based on selected correlation time τ. When the amount of data was large, the consumed time was 1/186 that of the ordinary DAVAR algorithm, which greatly reduced the calculation time. Reference [10] proposed a time-varying window dynamic Allan variance method based on fuzzy control. Experimental results showed that the method could effectively identify the characteristics of fiber optic gyroscopic dynamic signals, and the performance evaluation index improved by at least 30%. Reference [11] improved the dynamic Allan variance by establishing a dynamic noise model and using a new truncation window based on entropy features. The results showed that the dynamic Allan variance could extract noise features well.

In order to solve the problems of low accuracy, long data processing time, and poor tracking ability of noise characteristics when using the traditional DAVAR method to analyze MEMS gyro’s noise, a PID-DAVAR adaptive algorithm combining PID principle and DAVAR is designed. The gyro’s angular rate signal is dynamically intercepted in real-time, and the length of the truncation window is adaptively adjusted according to the characteristics of the gyro’s output signal. The adaptive adjustment of window size not only meets the requirement of variance confidence, but also shortens the running time owning good signal tracking ability, and high efficiency.

This article is organized as follows. In Section 2, principles of Allan variance and the character analysis of five random noise terms are introduced. In Section 3, principles of dynamic Allan variance are introduced in detail. In Section 4, by introducing the PID principle, the PID-DAVAR adaptive algorithm is proposed, and the calculation process of the algorithm is gradually introduced. In Section 5, the effectiveness of the PID-DAVAR adaptive algorithm is verified by vibration experiments. The conclusion is given in Section 6.

## 2. Allan Variance Principle

### 2.1. Principle of Conventional Allan Variance

Allan variance is a time-domain analysis method proposed by David Allan of the National Bureau of Standards (NBS) in the mid-1960s as a single data analysis method, can also be added as a frequency domain analysis technique [12]. It can analyze the gyro parameters and identify error terms, calculate and characterize all kinds of noise in the output signal of MEMS gyroscope [13,14].

The sampling period of the MEMS gyroscope is set as $\tau_{0}$, a total of N angular rate data were sampled, and N data were evenly divided into D(D=N/m) groups, each consisting of m(m≤(N−1)/2) data. The average value of each group is:

(1) $${\bar{\omega }}_{i}\left(m\right)=\frac{1}{m}\sum_{j=1}^{m}\omega_{\left(i-1\right)m+j},\left(i=1,2,\cdots ,D\right)$$

The estimation formula of Allan variance [15,16] is:

(2) $$\sigma^{2}\left(\tau \right)=\frac{1}{2}\langle {\left({\bar{\omega }}_{i+1}\left(m\right)-{\bar{\omega }}_{i}\left(m\right)\right)}^{2}\rangle =\frac{1}{2(D-1)}\sum_{i=1}^{D-1}{\left[{\bar{\omega }}_{i+1}\left(m\right)-{\bar{\omega }}_{i}\left(m\right)\right]}^{2}$$

where 〈 〉 represents the overall average of the data, the correlation time is $\tau =m\tau_{0}$, ${\bar{\omega }}_{i}\left(m\right)$ is the average angular rate of the group i, and i is the grouping number (i=1,2,⋯,D−1).

### 2.2. Characterization of Five Typical Random Noise Terms

The error of MEMS gyroscope is generally divided into deterministic error and random error. The deterministic error is compensated by experimental calibration, while the random error is accidental and random, which changes with time. To reduce the influence of random error on the gyroscope, the noise characteristics are identified by Allan variance, and the software algorithm is used to compensate, so as to improve the accuracy of the gyroscope [17,18]. Typical MEMS gyroscope random errors include quantization noise (QN), angular random walk (ARW), bias instability (BI), rate random walk (RRW), and rate ramp (RR) [19]. The relationship between these five types of noise terms and Allan variance is derived in Equations (3)–(7).

1. Quantization noise refers to a high-frequency noise generated during the conversion of digital signals to analog signals. The Allan variance is expressed as:
  (3) $$\sigma_{QN}^{2}\left(\tau \right)=\frac{\sqrt{3}Q}{\tau }$$
2. Angle random walk is high-frequency noise caused by MEMS gyro angular rate random white noise integration. The Allan variance is expressed as:
  (4) $$\sigma_{ARW}^{2}\left(\tau \right)=\frac{N^{2}}{\tau }$$
3. Bias instability refers to the low-frequency bias drift caused by the flicker noise of electronic circuits, environmental noise, and other components. The Allan variance is expressed as:
  (5) $$\sigma_{BI}^{2}\left(\tau \right)=\frac{2B^{2}ln2}{\pi }\approx {\left(\frac{B}{0.6648}\right)}^{2}$$
4. Rate random walk refers to the random error generated by integrating the power spectral density of the bandwidth angular acceleration signal. The Allan variance is expressed as:
  (6) $$\sigma_{RRW}^{2}\left(\tau \right)=\frac{K^{2}\tau }{3}$$
5. Rate ramp refers to the extremely slow monotonic change of the MEMS gyroscope during the long-term output process. The Allan variance is expressed as:
  (7) $$\sigma_{RR}^{2}=\frac{R^{2}\tau^{2}}{2}$$

Assuming that these five random error sources are independent of each other, the total Allan variance is the sum of the variances of various error sources, namely:

(8) $$\sigma_{all}^{2}=\frac{3Q^{2}}{\tau^{2}}+\frac{N^{2}}{\tau }+{\left(\frac{B}{0.6643}\right)}^{2}+\frac{K\tau^{2}}{3}+\frac{R^{2}\tau^{2}}{2}$$

Equation (3) is fitted by the least square method, and the total Allan standard deviation $\sigma_{all}$ is:

(9) $$\sigma_{all}\approx \sum_{i=-2}^{2}A_{i}\tau^{i/2}$$

According to the fitting coefficient, the parameter estimation can be obtained, as shown in Table 1.

## 3. Principle of DAVAR

**Figure 1:**
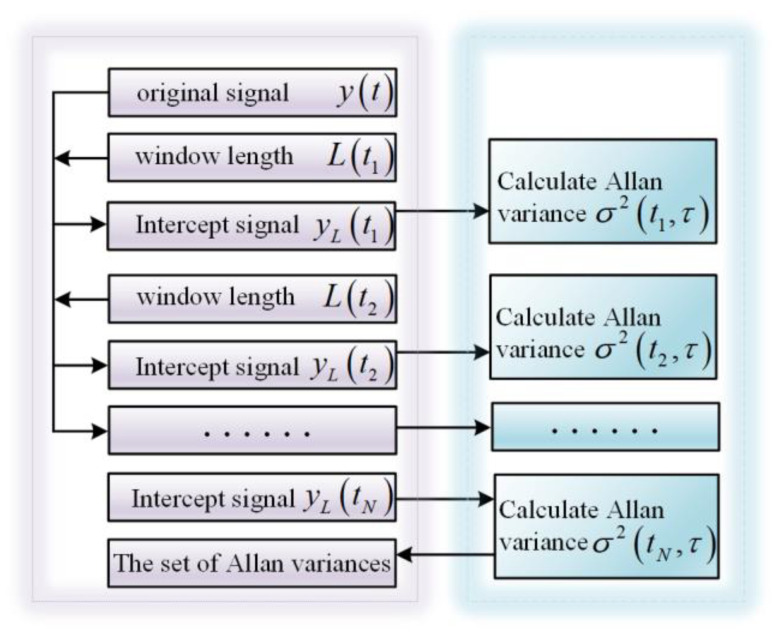
DAVAR algorithm flow char.

The conventional Allan variance is generally used to analyze the stability of the error signal [20], which is suitable for the ideal time-varying signal analysis and process. However, in the actual inertial navigation application environment, the signal shows obvious instability in a very short time. To accurately analyze the random noise in the MEMS gyroscope’s signal, the DAVAR method is used to analyze the gyroscope’s output signal, and accurately represent the real-time characteristics of the MEMS gyroscope’s random noise [21,22]. The specific steps are shown in Figure 1, and described as follows:

(1) Fix an analysis point, let $t=t_{1}$;
(2) Take the analysis point $t_{1}$ as the center, the fixed length $L\left(t_{1}\right)$ is selected to intercept the original output signal;
(3) Take the signal intercepted in step (2) as the research object, the Allan variance $\sigma^{2}\left(t_{1},\tau \right)$ is calculated;
(4) Continue to select another time analysis point, namely $t=t_{2}$. The selection of $t_{2}$ should make the intercepted signal data overlap with the intercepted data of the previous time analysis point $t_{1}$, repeat steps (2)~(4) to obtain Allan variance $\sigma^{2}\left(t_{2},\tau \right)$. Analogously, piecewise estimation is performed through a moving window, and the Allan variance set $\sigma^{2}\left(t_{N},\tau \right)$ is obtained by multiple calculations;
(5) The Allan variance set $\sigma^{2}\left(t_{1},\tau \right),\cdots ,\sigma^{2}\left(t_{N},\tau \right)$ are arranged in chronological order, which corresponds to different time analysis points t and different interception intervals τ. It is reflected in the form of a 3D graph, which characterizes the stability of real-time measurement of the MEMS gyroscope’s signal.

Considering the continuous situation, assuming that y(t) is the angular rate data collected by the MEMS gyroscope, the output signal $y\left(t^{\prime }\right)$ is intercepted in the window interval $\left(t-L/2\right)\leq t^{\prime }\leq \left(t+L/2\right)$, and the truncated signal is expressed in mathematical form as:

(10) $$y_{L}\left(t,t^{\prime }\right)=y\left(t^{\prime }\right)P_{L}\left(t-t^{\prime }\right)$$

where, $y_{L}\left(t,t^{\prime }\right)$ is truncated data of MEMS gyro’s signal. L is the length of the truncation window, it is assumed to be odd. $P_{L}\left(t\right)$ is the function of truncation window length, as shown in Equation (11).

(11) $$P_{L}\left(t\right)=\left\{ \begin{array}{l} 1\begin{array}{cc} , & \left|t\right|\leq L/2 \end{array}\\ 0\begin{array}{cc} , & \mathrm{else} \end{array} \end{array}\right.$$

No matter which window form is selected, t always represents the center of the rectangular window. By convoluting truncated data $y_{L}\left(t,t^{\prime }\right)$ and the Allan window $A_{\tau }\left(t^{\prime }\right)$, a continuous growth process is constructed. It can be written as:

(12) $$\Delta \left(t,t^{\prime },\tau \right)=A_{\tau }\left(t^{\prime }\right)*y_{L}\left(t,t^{\prime }\right)=\int_{-\infty }^{+\infty }A_{\tau }\left(t^{\prime }-t^{\prime \prime }\right)y_{L}\left(t,t^{\prime \prime }\right)dt^{\prime \prime }$$

where, the star sign stands for convolution, and $A_{\tau }\left(t^{\prime }\right)$ is the Allan window, defined as:

(13) $$A_{\tau }\left(t^{\prime }\right)=\left\{ \begin{array}{l} -\frac{1}{\tau }\begin{array}{cc} , & 0\leq t^{\prime }\leq \tau ; \end{array}\\ \frac{1}{\tau }\begin{array}{cc} , & -\tau \leq t^{\prime }<0; \end{array} \end{array}\right.$$

where the variables in Equation (13) need to meet the conditions: $t-\left(L/2-\tau \right)\leq t^{\prime }\leq t+\left(L/2-\tau \right)$, $0<\tau \leq \tau_{\max}$.

$\tau_{\max}$ is the maximum observation interval. Here, we can set $\tau_{\max}$ as:

(14) $$\tau_{\max}=\left\lfloor \frac{L}{3}\right\rfloor$$

In Equation (14), ⌊ ⌋ is the symbol that is rounded down to the nearest integer. Substituting Equation (12) into Equation (2), it can be written as:

(15) $${\hat{\sigma }}_{y}^{2}\left(t,\tau \right)=\frac{1}{2}\langle \Delta^{2}\left(t,t^{\prime },\tau \right)\rangle =\frac{1}{2\left(L-2\tau \right)}\int_{t-L/2+\tau }^{t+L/2-\tau }\Delta^{2}\left(t,t^{\prime },\tau \right)dt^{\prime }$$

The dynamic Allan variance is the expected value of Equation (15), that is:

(16) $$\sigma_{DAVAR}^{2}\left(t,\tau \right)=E\left[\sigma_{y}^{2}\left(t,\tau \right)\right]=\frac{1}{2}E\left[\langle \Delta^{2}\left(t,t^{\prime },\tau \right)\rangle \right]$$

## 4. Dynamic Allan Variance Based on Adaptive PID Principle

### 4.1. PID Principle in PID-DAVAR Adaptive Algorithm

The traditional DAVAR method used a rectangular window with a fixed window length to intercept the output signal of the MEMS gyroscope. However, DAVAR with a fixed window length is difficult to meet the requirements of MEMS gyroscope’s signal accuracy identification in the whole time domain. If the data intercepted by the window is long, the dynamic tracking ability of the DAVAR method will be greatly weakened, resulting in a small gap with the Allan variance, which cannot represent the characteristics of random noise in detail and is insensitive to the tracking of burst signals; If the data intercepted by the window is short, although it can achieve good tracking effect, it will lose a certain confidence of variance, resulting in a large analysis error [23,24]. Therefore, a method of adjusting the length of the DAVAR window based on the adaptive PID principle is proposed. The length of the truncation window is adjusted adaptively to achieve better tracking ability and ideal variance confidence [25,26].

PID is a linear combination of the proportion (P), integral (I), and differential (D) of the error in the feedback system. The error e(t) is obtained by subtracting the input value x(t) from the actual output value y(t). It can be expressed as:

(17) e(t)=x(t)−y(t)

The ideal PID principle can be expressed as:

(18) $$u\left(t\right)=K_{P}\left[e\left(t\right)+\frac{1}{T_{I}}\int e\left(t\right)dt+T_{D}\frac{de\left(t\right)}{dt}\right]$$

where, u(t) is the output value at a time t, $K_{P}$ is the proportional coefficient, $T_{I}$ is the integral time constant, and $T_{D}$ is the differential time constant. The error e(t) is used as the input and u is used as the output.

Because Equation (18) contains mathematical algorithms such as integral and differential, it is difficult to program in the computer control system, so it needs to be transformed into a discrete control algorithm. Therefore, Equation (18) is transformed into a transfer function form:

(19) $$G\left(s\right)=\frac{U\left(s\right)}{E\left(s\right)}=K_{P}\left(1+\frac{1}{T_{I}s}+T_{D}s\right)$$

Equation (18) is discretized, and many sampling time points KT are used instead of continuous time t. The integral part is added, and the differential part is expressed by increment. Let t≈kT, so that:

(20) $$\int_{0}^{t}e\left(t\right)dt\approx T\sum_{j=0}^{k}e\left(jT\right)=T\sum_{j=0}^{k}e\left(j\right)$$

(21) $$\frac{de\left(t\right)}{dt}\approx \frac{e\left(kT\right)-e\left(\left(k-1\right)T\right)}{T}=\frac{e\left(k\right)-e\left(k-1\right)}{T}$$

Among them, k(k=0,1,2,3,…) is the sampling time point. *T* is the sampling period. In order to represent clarity and reduce the calculation time, the value of T is reduced, and then e(kT)≈e(k) in Equation (21).

Substitute Equation (20) and Equation (21) into Equation (18) to get:

(22) $$\left\{ \begin{array}{l} E\left(k\right)=\left[e\left(k\right)-e\left(k-1\right)\right]\\ u\left(k\right)=K_{P}\left[e\left(k\right)+\frac{T}{T_{I}}\sum_{j=0}^{k}e\left(j\right)+\frac{T_{D}}{T}E\left(k\right)\right] \end{array}\right.$$

Simplifying Equation (22) can be redescribed as:

(23) $$\left\{ \begin{array}{l} E\left(k\right)=\left[e\left(k\right)-e\left(k-1\right)\right]\\ u\left(k\right)=K_{P}e\left(k\right)+K_{I}\sum_{j=0}^{k}e\left(j\right)+K_{D}E\left(k\right) \end{array}\right.$$

Equation (23) can also be called position PID control. Where, u(k) is the output of the *k*th sampling, e(k) is the error of the *k*th sampling, e(k−1) is the error of the (*k* − 1)th sampling, $K_{I}=K_{P}\frac{T}{T_{I}}$ is the integral coefficient, and $K_{D}=K_{P}\frac{T_{D}}{T}$ is the differential coefficient.

It can be seen from Equation (23) that the biggest disadvantage of using this algorithm to program is that each output of the system is closely related to the cumulative error at all times. Each calculation needs to accumulate an error at each time, resulting in a large amount of calculation and waste of computing resources. In order to avoid the above problems, using incremental PID algorithm, it can be seen from Equation (23):

(24) $$\left\{ \begin{array}{l} E\left(k-1\right)=\left[e\left(k-1\right)-e\left(k-2\right)\right]\\ u\left(k-1\right)=K_{P}e\left(k-1\right)+K_{I}\sum_{j=0}^{k}e\left(j\right)+K_{D}E\left(k-1\right) \end{array}\right.$$

By subtracting Equation (23) from Equation (24):

(25) $$\Delta u\left(k\right)=K_{p}\left[e\left(k\right)-e\left(k-1\right)\right]+K_{I}e\left(k\right)+K_{D}\left[e\left(k\right)-2e\left(k-1\right)+e\left(k-2\right)\right]$$

where, Δu(k) is the increment of the output value at the *k*th sampling, and e(k−2) is the error at the (*k* − 2)th sampling. The incremental PID algorithm does not need cumulative calculation and only needs one calculation cycle to correct the error. The whole system has strong fault tolerance, convenient implementation, and wide application.

### 4.2. PID-DAVAR Adaptive Algorithm

In order to better analyze the dynamic characteristics of the MEMS gyroscope’s random noise [27], the PID-DAVAR adaptive algorithm is proposed. It can automatically adjust the length of the truncation window according to the fluctuation of the MEMS gyro’s signal [28]. The adaptive calculation is performed every $t_{0}$ time. The deviation index ε of the data needs to be calculated in this truncation window before PID-DAVAR adaptive algorithm.

(26) $$\varepsilon =\sigma -d_{ave}$$

where, σ is the standard deviation of the data in the truncation window, $d_{ave}$ and is the average value of the data in the truncation window.

**Figure 2:**
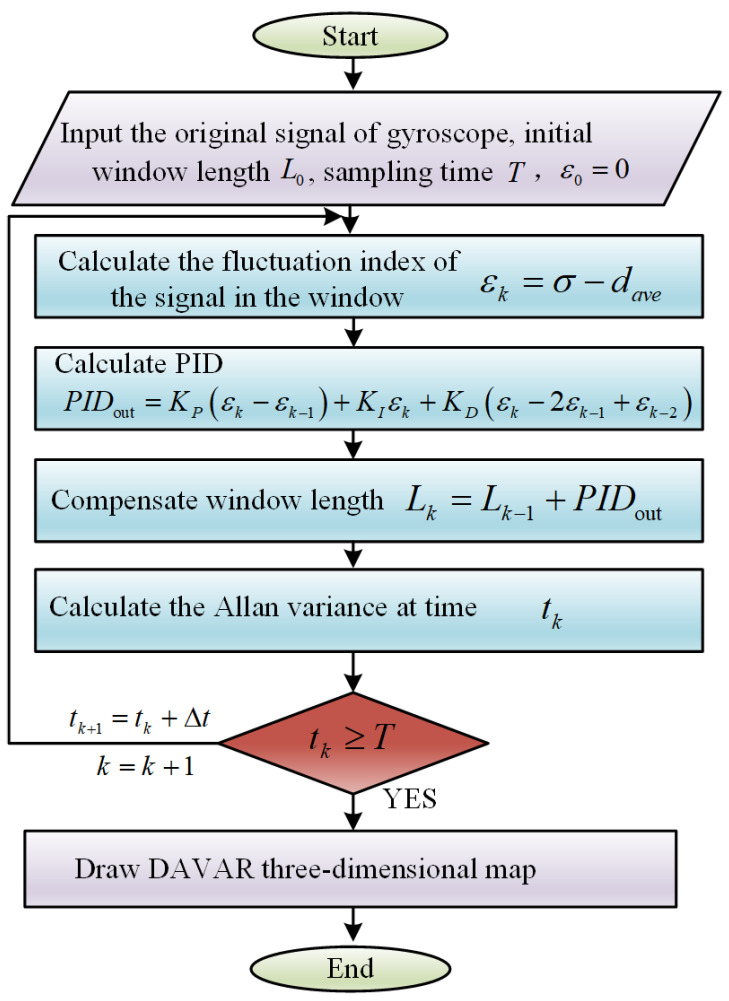
PID-DAVAR adaptive algorithm flow chart.

The specific flow chart is shown in Figure 2. The premise of the PID-DAVAR adaptive algorithm is to input the measured MEMS gyroscope original signal, sampling time *T*, set the initial window length $L_{0}$, and the initial deviation index $\varepsilon_{0}=0$. The deviation index $\varepsilon_{k}$ is calculated by Equation (26). The deviation index $\varepsilon_{k}$ can be regarded as e(k) in Equation (25). The difference between $\varepsilon_{k}$ and $\varepsilon_{k-1}$ is multiplied by $K_{p}$ as the proportional term of the output of the PID-DAVAR adaptive algorithm. The integral term and differential term are also replaced by the deviation index $\varepsilon_{k}$. The output of the PID-DAVAR adaptive algorithm is added to the previous window length $L_{k-1}$ to obtain the current window number $L_{k}$. According to the length of current windows $L_{k}$, the Allan variance at $t_{k}$ can be calculated. Δt is the time interval between two adjacent truncation windows. When $t_{k}<T$ the $t_{k}$ is shifted by Δt and continues to enter the calculation cycle of Allan variance. When $t_{k}\geq T$ all the calculated Allan curves are superimposed in chronological to obtain the DAVAR 3D diagram.

When the fluctuation deviation index ε is large, the output data of the MEMS gyroscope fluctuates violently. At this time, the length of the truncation window is reduced, the instability of the data is accurately characterized, and a better tracking effect is achieved. When the fluctuation deviation index ε is small, the output data of the MEMS gyroscope is stable. At this time, the length of the truncation window should be increased, which not only highlights the stationary characteristics of the data but also ensures the variance confidence of DAVAR. This algorithm can adaptively adjust the length of the window according to the fluctuation characteristics of the data, realizing the real-time tracking of unstable data and ensuring the variance confidence of DAVAR. Meanwhile, it also reduces the amount of calculation, shortening the running time and improving computational efficiency.

## 5. Experimental

**Figure 3:**
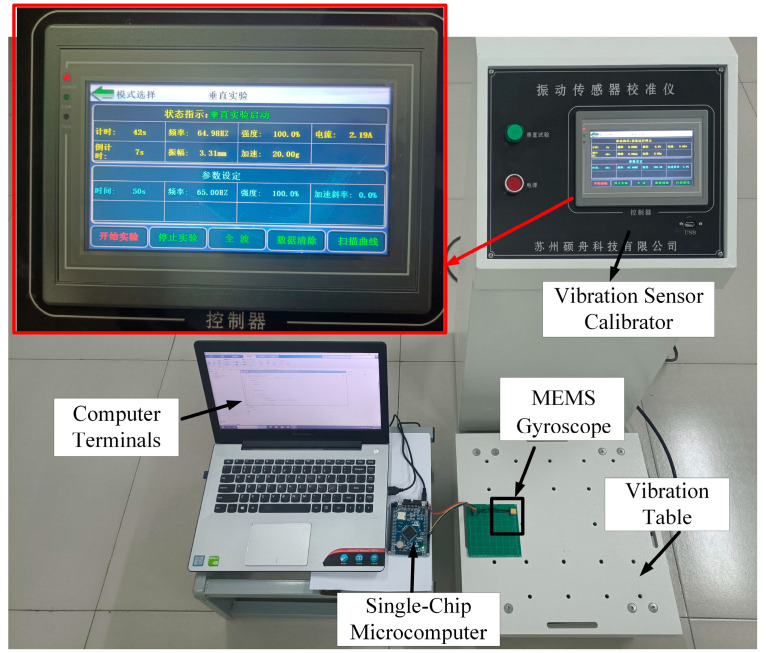
Vibration test device diagram.

**Figure 4:**
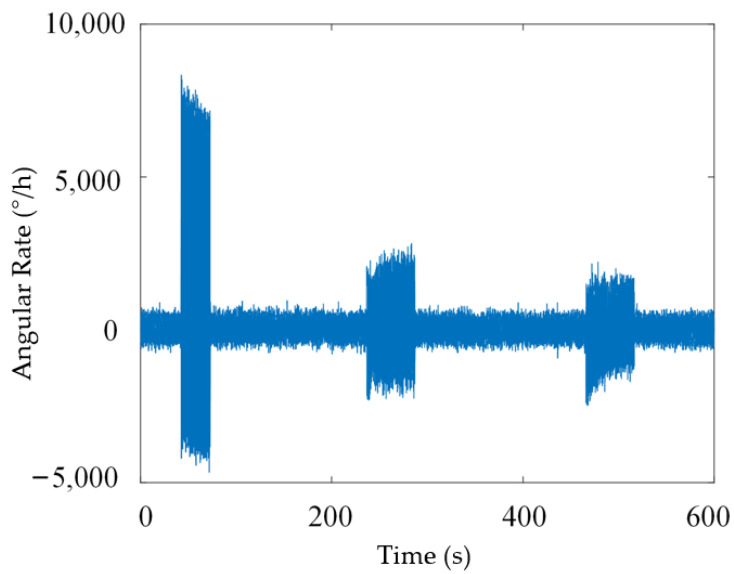
MEMS gyroscope original output signal.

In order to verify the PID-DAVAR adaptive algorithm, experiments were performed at a room temperature of 25 °C. The MEMS gyroscope with the model MGZ211HC was placed on the vibration tester platform to collect the dynamic output signal. The STM32F2103MCU communicated with the MGZ211HC gyroscope through full-duplex SPI communication. The sampling frequency was set to 100 Hz and the sampling interval was 0.01 s. In order to ensure the integrity of the data, the data was continuously collected and sent to the PC for recording in real-time by USART. The experimental device and output signal are shown in Figure 3 and Figure 4.

**Figure 5:**
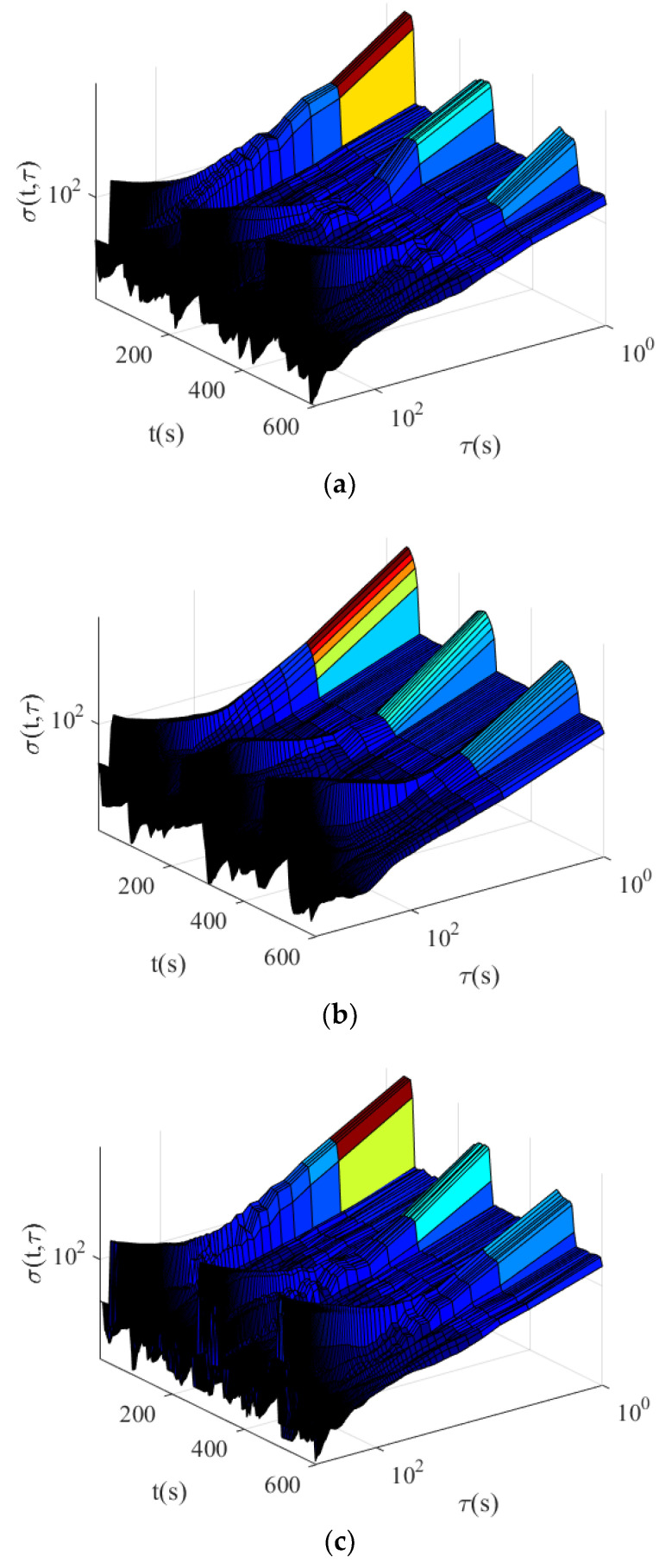
DAVAR results for different window lengths. (**a**) the window length of 1001; (**b**) the window length of 3001; (**c**) the window length of adaptive.

The MEMS gyroscope’s original output signal is shown in Figure 4. The vibration platform starts to vibrate at t = 40 s, the vibration frequency is 60 Hz, and the duration is 30 s. The second and third vibrations started at t = 230 s and t = 460 s, respectively. The vibration frequencies are 65 Hz and 70 Hz, and the duration times are both 50 s. Then, DAVAR is used to analyze the vibration data, and the length of the truncation window is set to *L* = 1001 and *L* = 3001, the traditional DAVAR method and the PID-DAVAR adaptive algorithm are analyzed respectively. The results are shown in Figure 5.

As can be seen in Figure 5, the MEMS gyroscope’s output signal behaves differently for varying window lengths. When the truncation window of length *L* = 1001, the data fluctuation is not stable, and the curve has more inflection points, the difference between the maximum and minimum values of *Z*-axis data fluctuation is 418.28. When the truncation window of length *L* = 3001, the fluctuation of the data becomes stable and the curve is smooth, the difference between the maximum and minimum values of *Z*-axis data fluctuation is 275.19. When the length of the truncation window is adaptive, the output signal of the MEMS gyroscope changes drastically under vibration. In the absence of vibration, the signal is stable and the curve is smooth, the difference between the maximum and minimum values of *Z*-axis data fluctuations is 386.71.

**Figure 6:**
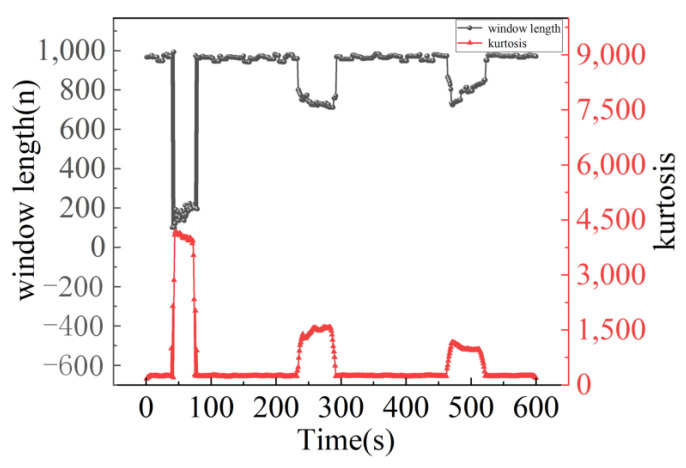
Comparison between kurtosis change and adaptive window length change.

As the length of the truncation window is adaptively adjusted, the number of windows changes with the DAVAR calculation process as shown in Figure 6. Kurtosis is used to represent the degree of fluctuation of data. At t = 41 s–76 s, t = 234 s–291 s, and t = 63 s–519 s, the length of the truncation window gradually decreases with the fluctuation of the kurtosis. The larger the kurtosis value, the smaller the corresponding truncation window length to ensure the dynamic tracking effect of the gyroscope’s signal. By automatically adjusting the length of the truncation window, the algorithm accurately and clearly shows the unstable characteristics of the MEMS gyroscope’s signal, shortens the running time, and improves the operating efficiency.

Taking the data in t = 234 s–291 s as an example, the tracking ability and data processing time of the fixed window and adaptive window are compared in Table 2. The CPU model used in this paper is Intel (R) Core (TM) i3-10100F, and the RAM of the machine is 32.0 GB.

It can be seen from Table 2 that the starting point of data mutation under the PID-DAVAR adaptive algorithm is t = 235.5 s, which is closest to the mutation reference value t = 237.3 s. It shows that the PID-DAVAR adaptive algorithm has a better tracking ability for abrupt signals than fixed window DAVAR. The total time is 8.65 s, which is nearly 10 times less than the fixed window DAVAR. It can be seen that the PID-DAVAR adaptive algorithm can not only characterize the fluctuation and mutation characteristics of MEMS gyroscope signals but also shorten the time of data processing.

**Figure 7:**
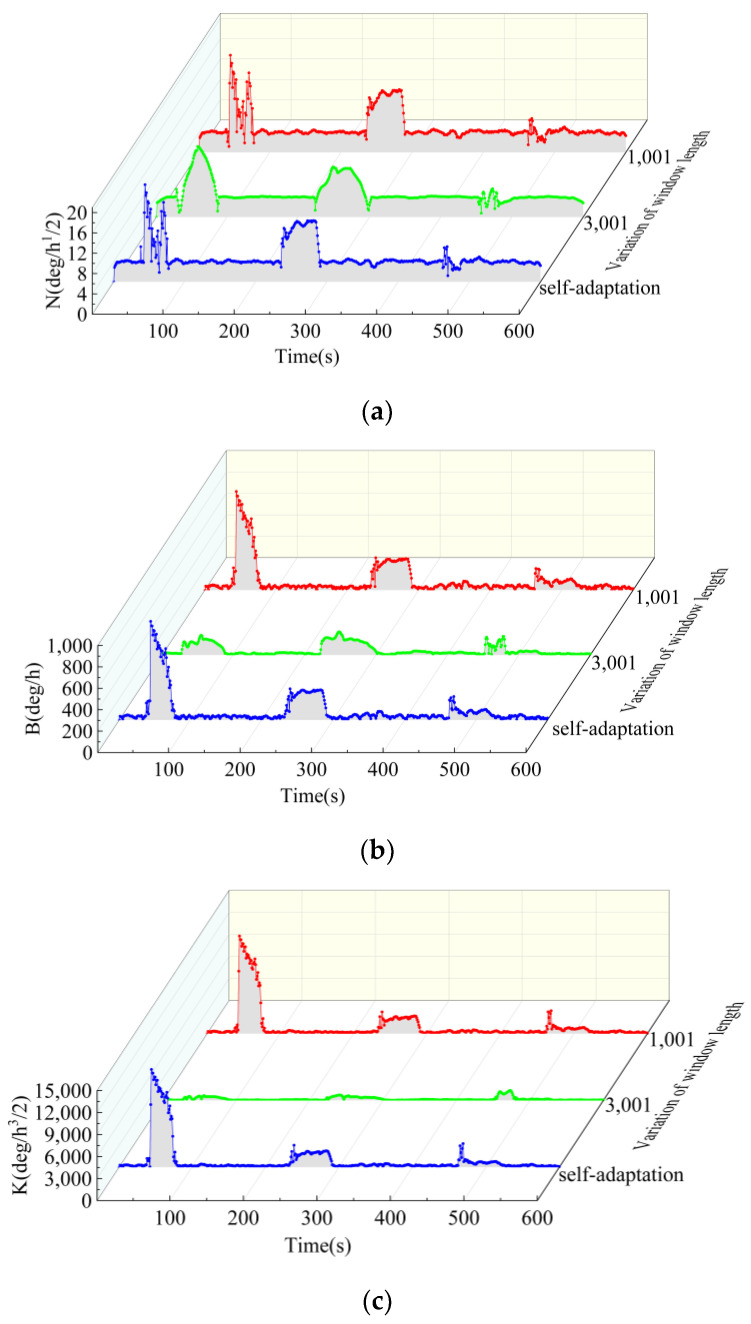
DAVAR analysis of noise coefficient under different window lengths. (**a**) Change curve of ARW coefficient N under different window lengths; (**b**) Change curve of BI coefficient B under different window lengths; (**c**) Change curve of RRW coefficient K under different window lengths.

In order to verify the tracking ability of the PID-DAVAR adaptive algorithm to random error, angle random walk, bias instability, and rate random walk are selected for identification analysis. Figure 7 shows the variation curves of noise coefficients under different window lengths.

As the gyroscope is in a static state (t = 0–40 s, t = 77–233 s, t = 292–465 s, t = 520–600 s) in Figure 7, the coefficient of each noise is small without obvious fluctuation. As the shaker starts to vibrate at 60 Hz, 65 Hz, and 70 Hz (t = 41–76 s, t = 234–291 s, t = 463–519 s), the noise terms of the gyroscope change significantly. When the shaker stops vibrating, all the noise terms of the gyroscope are restored to the previous state. When *L* = 1001, the three noise coefficients of the gyroscope change significantly under dynamic conditions, and it is difficult to accurately locate the mutation point of the signal. When *L* = 3001, the three noise coefficient curves of the gyroscope are relatively smooth, and it is difficult to determine the specific location of the mutation. Using the PID-DAVAR adaptive algorithm, the mutation trend is slow and the steady state can be restored at the fastest speed. The algorithm can not only clearly obtain the position of the mutation point, but also accurately express the variation characteristics of the noise coefficient under dynamic conditions.

The angular rate signal of MGZ211HC gyro acquired at room temperature and stationary state is calculated by Allan variance. The calculated angular random walk, bias instability, and rate random walk are used as reference values. In order to verify the tracking ability and variance confidence of the PID-DAVAR adaptive algorithm, 100 sets of sample data are selected to calculate the average value of the three noise coefficients, which are compared with the reference values. The comparison results are shown in Table 3.

When *L* = 1001, the length of the truncation window is fixed to 1001 data. At this time, fewer data are selected, which leads to obvious changes in the coefficients of angular random walk, bias instability, and rate random walk. The values are: 2.01$\left({}^{\circ}\right)\cdot h^{-1/2}$, 14.71$\left({}^{\circ}\right)\cdot h^{-1}$, and 91.04$\left({}^{\circ}\right)\cdot h^{-3/2}$. These three coefficients have large errors compared with the standard values. Therefore, the setting of this window length does not guarantee the MEMS gyro variance confidence. When *L* = 3001, the length of the truncation window is fixed to 3001 at this time. Due to the large number of selected data, the angle random walk, bias instability, and rate random walk coefficients do not change significantly, and the values become smaller, which are 1.99$\left({}^{\circ}\right)\cdot h^{-1/2}$, 7.86$\left({}^{\circ}\right)\cdot h^{-1}$, and 34.07$\left({}^{\circ}\right)\cdot h^{-3/2}$, respectively. Compared with the reference value, the error between the two is larger and the calculation time is prolonged. Therefore, the setting of this window length cannot accurately represent the dynamic characteristics of the MEMS gyroscope’s random noise. When the window length is adaptive, the length of the truncation window is variable. The larger the fluctuation of the data, the shorter the window length, and vice versa. The coefficients of angular random walk, bias instability, and rate random walk are closer to the reference value. Their values are 1.79$\left({}^{\circ}\right)\cdot h^{-1/2}$, 15.32$\left({}^{\circ}\right)\cdot h^{-1}$, and 95.76$\left({}^{\circ}\right)\cdot h^{-3/2}$, which differ from the reference value by 0.07$\left({}^{\circ}\right)\cdot h^{-1/2}$, 3.57$\left({}^{\circ}\right)\cdot h^{-1}$, and 6.62$\left({}^{\circ}\right)\cdot h^{-3/2}$ respectively. The errors are 4%, 19%, and 6% respectively, and the average error is about 10%. By using adaptive window length based on the adaptive PID principle, PID-DAVAR adaptive algorithm can describe the dynamic characteristics of MEMS gyro’s random noise precisely with fewer computing resources, ensuring the confidence of variance and high efficiency.

## 6. Conclusions

Random noise is a key factor affecting the performance improvement of the MEMS gyroscope. It is necessary to dynamically analyze and identify various noise items in MEMS gyroscope. In order to dynamically analyze the random noise characteristics of the MEMS gyroscope, PID-DAVAR adaptive algorithm is proposed in this paper. The PID-DAVER adaptive algorithm combines the PID principle and the DAVAR method to realize the adaptive adjustment of the truncation window’s length. The algorithm can adjust the length of the truncation window according to the size of the data fluctuation kurtosis. The larger the kurtosis is, the shorter the length of the truncation window, and the better the tracking effect of the abrupt signal. The smaller the kurtosis is, the longer the truncation window length is, and the signal is stable and the curve is smooth. According to the experimental results, the noise coefficient calculated by the PID-DAVAR adaptive algorithm is more accurate than the traditional DAVAR method. The average error of the coefficients of angular random walk, bias instability, and rate random walk is only 10%, and the minimum coefficient error reaches 4%. It only takes 8.65 s to calculate and draw the DAVAR three-dimensional diagram of 60,000 data. The algorithm saves a lot of computing resources and shortens the calculation time. It is beneficial to realize real-time online analysis and error compensation of MEMS gyro dynamic noise. The PID-DAVAR adaptive algorithm effectively guarantees the variance confidence of the MEMS gyroscope’s signal processing and improves the tracking ability of signal mutation. In practical applications, especially when inertial navigation devices such as MEMS gyroscopes and fiber optic gyroscopes work in harsh environments, the PID-DAVAR adaptive algorithm provides further technical support for the dynamic analysis of gyro random error terms. When the amount of data to be processed is large or the power storage of mobile devices is small, this method can also play a certain role in the dynamic analysis of gyro error terms.

## Figures and Tables

**Table 1:** Parameter estimation and slope of noise term.

Main Noise Terms	Parameter Estimation	Slope Value
Quantization Noise	$A_{-2}/\sqrt{3}$	−1
Angular Random Walk	$A_{-1}$	−1/2
Bias Instability	$1.505A_{0}$	0
Rate Random Walk	$\sqrt{3}A_{1}$	1/2
Rate Ramp	$\sqrt{2}A_{2}$	1

**Table 2:** Mutation Point and Total Time Comparison Table.

Window Length	Mutation Start Point (s)	Mutation End Point (s)	Total Time (s)
Mutation Reference Value	237.3	286.3	
1001	231.6	289.7	20.87
3001	218.8	296.5	89.46
Adaptive Window	235.5	285.4	8.65

**Table 3:** Noise coefficients on different window lengths.

**Title 1**	$N/\left({}^{\circ}\right)\cdot h^{-1/2}$	$B/\left({}^{\circ}\right)\cdot h^{-1}$	$K/\left({}^{\circ}\right)\cdot h^{-3/2}$
Reference Value	1.72	18.89	102.28
1001	2.01	14.71	91.04
3001	1.99	7.86	34.07
Self-Adaptation	1.79	15.32	95.76
